# Responding to non-communicable diseases in Zambia: a policy analysis

**DOI:** 10.1186/s12961-017-0195-7

**Published:** 2017-04-24

**Authors:** Mulenga M. Mukanu, Joseph Mumba Zulu, Chrispin Mweemba, Wilbroad Mutale

**Affiliations:** The University of Zambia, School of Public Health, P.O. Box 50110, Lusaka, Zambia

**Keywords:** Health policy, Policy analysis, Non-communicable diseases, Zambia

## Abstract

**Background:**

Non-communicable diseases (NCDs) are an emerging global health concern. Reports have shown that, in Zambia, NCDs are also an emerging problem and the government has begun initiating a policy response. The present study explores the policy response to NCDs by the Ministry of Health in Zambia using the policy triangle framework of Walt and Gilson.

**Methods:**

A qualitative approach was used for the study. Data collected through key informant interviews with stakeholders who were involved in the NCD health policy development process as well as review of key planning and policy documents were analysed using thematic analysis.

**Results:**

The government’s policy response was as a result of international strategies from WHO, evidence of increasing disease burden from NCDs and pressure from interest groups. The government developed the NCD strategic plan based on the WHO Global Action Plan for NCDs 2013–2030. Development of the NCD strategic plan was driven by the government through the Ministry of Health, who set the agenda and adopted the final document. Stakeholders participated in the fine tuning of the draft document from the Ministry of Health. The policy development process was lengthy and this affected consistency in composition of the stakeholders and policy development momentum. Lack of representative research evidence for some prioritised NCDs and use of generic targets and indicators resulted in the NCD strategic plan being inadequate for the Zambian context. The interventions in the strategic plan also underutilised the potential of preventing NCDs through health education. Recent government pronouncements were also seen to be conflicting the risk factor reduction strategies outlined in the NCD strategic plan.

**Conclusion:**

The content of the NCD strategic plan inadequately covered all the major NCDs in Zambia. Although contextual factors like international strategies and commitments are crucial catalysts to policy development, there is need for domestication of international guidelines and frameworks to match the disease burden, resources and capacities in the local context if policy measures are to be comprehensive, relevant and measurable. Such domestication should be guided by representative local research evidence.

## Background

Non communicable diseases (NCDs) have increasingly been contributing to the global disease burden, especially in low- and middle-income countries (LMICs) [1, 2]. In such countries, there is evidence that a significant proportion of the prevalence of the common NCDs like diabetes and cardiovascular diseases (CVDs) is now occurring in the productive age group of those aged between 30 and 60 years [3, 4]. In Zambia, there have been no national representative population-based studies to determine the burden of NCDs. Studies that have been conducted found that the prevalence of hypertension in adults ranged between 25.8% and 32.8% [5, 6] in rural areas and 34.8% in urban areas [7]. Although earlier studies estimated the prevalence of diabetes to be 4.6% for adult females and 5.35% for adult males in Lusaka [8], a recent study estimated a lower prevalence of 3.5% in Zambian communities [9]. Recent WHO reports show that NCDs in 2016 contributed to 33% of deaths for all ages in Zambia [10], as compared to 23% in 2014 [4].

In response to the increasing burden being evidenced by many countries, there have been several efforts on the global arena to address NCDs in recent years. In 2000, the WHO produced the global strategy for the prevention and control of NCDs, which was followed by an action plan in 2008 [11]. In addition, the WHO also developed global strategies for specific risk factors like diet, physical activity and alcohol [12]. The global attention on NCDs culminated in a high level meeting to discuss NCDs held during the 66th General Assembly meeting of the United Nations (UN) in 2011 [13]. Arising from this high level meeting was the Political Declaration on NCDs, which tasked countries to begin taking action against the threatening epidemic of NCDs. Zambia, like most members of the UN, was a signatory to this declaration.

The 2011 Political Declaration, among other matters, encouraged countries to establish or support and strengthen multisectoral national policies that help reduce risk factors and create health promoting environments [14, 15]. Reviews of national responses to NCDs available in the literature show that most LMICs have developed strategies, programs, regulation and interventions that are both risk factor and disease specific [16]. Although the policy response to NCDs has been reported in studies in African countries like Tanzania [17], Mozambique [18], Ghana [19], Cameroun [20] and Zambia [21], the methodological approach used does not detail how these policies were developed, and yet, this is important if best practices are to be developed.

This study therefore aimed to contribute towards addressing this knowledge gap by analysing the policy response to non-communicable diseases by the Ministry of Health (MoH) in Zambia using the policy triangle framework of Walt and Gilson [22]. Using this framework, we aimed to identify the contextual factors that had shaped the NCD policies, to analyse the policy processes and actor involvement, and to identify and analyse the content of the available government policy provisions and strategies addressing NCDs.

## Methods

### Study design

A qualitative case study design was used for the study, with MoH headquarters as the primary unit of analysis. The ‘case’ was the national level health policy response to NCDs from 2008, when the Action Plan for the Global Strategy for the Prevention and Control of NCDs was produced by WHO, to 2015. A case study design was appropriate because it offered an in-depth exploration of the different aspects of the policy response to NCDs. In addition, case study designs have been recommended when performing policy analysis [23].

### Data collection

Data collection for the study was carried out between September and October 2015. The first step in data collection involved the review of policy documents. The documents reviewed were identified online through the MoH website, and then confirmed with the health policy unit from the MoH (Table 1). The second step involved conducting key informant interviews. The key informants targeted in this study were those that participated in the governments’ NCD policy development process. The first key informant was identified with assistance from the research unit at MoH. Snowballing was then utilised, asking each informant after the interview if they knew anyone else who would have information for the study. The key informants identified were then contacted either physically at their office or electronically through email or phone call and asked to participate in the study, and if they agreed, an appointment was set. A total of eight key informants participated in the study while two informants declined (Table 2).

**Table 1:** Documents reviewed for the study

Document/Report	Year/Period	Relevance to the study
Zambia across sector documents		
Vision 2030	2006	This document serves as a guide for all the development efforts of the country; as such, the goals and targets set in the vision determines the strategic focus in all economic sectors including health
Revised Sixth National Development Plan (R-SNDP)	2013–2016	This document is the main instrument for implementation of Government programs in the medium term in Zambia
Zambia health sector-specific documents		
National Health Policy	2013	This document states clear directions for the development of the Health Sector in Zambia; it sets out policy measures that are supposed to guide strategies and programs in the health sector
National Health Strategic Plan (NHSP)	2011–2016	It operationalises the national health policy in the medium term
Mid-term Review Report	2014	This document details the performance of the health sector according to the targets of the NHSP
Non-communicable disease (NCD)-specific documents		
National NCD strategic plan	2013–2016	It gives the strategic direction for NCDs, in the context of the broader health sector plans

**Table 2:** Key informants interviewed for the study

	Organisation	Involvement in non-communicable disease policy
Key informant #1	MoH	Policy development
Key informant #2	MoH	Policy development and implementation, Health promotion
Key informant #3	MoH	Specialist, policy implementation, cancer registry
Key informant #4	MCDMCH	Policy development and implementation
Key informant #5	WHO	Policy development, research
Key informant #6	WHO	Policy development, implementation
Key informant #7	Zambia Heart and Stroke Foundation	Advocacy, research
Key informant #8		Consultant, policy development

*MoH* Ministry of Health, *MCDMCH* Ministry of Community Development Mother and Child Health now Ministry of Community Development

### Data analysis

Thematic analysis approach using concept- and data-driven coding [24] was used for the study. Firstly, all the documents were read for familiarisation. Special attention was paid to the section in the documents that were addressing health at large and NCDs (if included) to establish the relevance of that document to the study. A brief summary (annotation) of the document was then made. After this, the document was read more critically to identify the key concepts (codes) in the documents. The key concepts identified were then categorised according to the broad idea that they represented. These categories were then analysed and grouped according to the predetermined themes from the policy analysis framework. The categories that were developed from the document analysis were the ones that were applied to the data from the data from the key informant interviews. However, if new concepts arose during the analysis of the transcripts, these were also noted and added to the existing categories. If they were not covered in the existing categories, new categories were established. Table 3 shows examples of the codes, categories and themes from the analysis.

**Table 3:** Selected codes, categories and themes from the data analysis

Codes	Categories	Themes
• Findings from local studies	Presence of evidence	Context
• Information from health facility		
• International reports		
• Responding to International resolutions	Global health agenda	
• Being part of global community		
• Need for development	National vision	
• Stakeholders involvement	Policy formulation process	Process: Policy development
• Developing the draft policy		
• Policy adoption		
• Political will		
• Lengthy policy development process	Challenges in the process	
• Lack of funding stalling the process		
• Influence from political powers		
• Contribution of stakeholder	Role of the stakeholders	Actors
• Drivers of the agenda	Influencers of policy	
• Situation analysis	Scope of the NCD policy	Content
• Prioritization of NCDs		
• Strategies to tackle NCDs		
• Health education not emphasized	Gaps in NCD policies	
• Lack of baseline data		
• Domestication of guidelines		

## Results

The components of the Walt and Gilson policy triangle framework [22] used in this study include context, process, actors and content. Below are the key findings according to this framework.

### Contextual factors in the policy development

Informants explained that certain happenings in the international arena dictate what direction the government has to take and what health priorities it will address. As such, the NCD policy was developed in response to global strategies mainly from WHO. For example, the NCD strategic plan was based on the guidelines from the WHO Global Action Plan for NCDs 2013–2030. Some informants stated that following global strategies was necessary as the country would then be able to compare its performance on NCDs with other countries around the globe, and not operate in a silo. One informant explained:

“*…Let me take you back a bit. In the year 2000, the United Nations met and came up with the global strategy for prevention and control of NCDs. Knowing very well that Zambia is part of the global village, so we had to do something about it…So after that global strategy was developed, the world again met in 2011, to make a political declaration on NCDs and Zambia was a signatory to that political declaration. Culminating out of that was the global NCD action plan 2013–2030 where a set of targets were set out. There are actually 9 voluntary targets. So based on those 9 voluntary targets, Zambia also had to act and come up with a strategic plan to sort of work out the activities on how to implement and meet those 9 voluntary targets*” (Key Informant 5)

In addition to the global drive, the availability of local data on the disease burden due to NCDs and the eminent epidemiological transition further compounded the need to start addressing these conditions on a national level. There also was a push from some interest groups, such as the Diabetic Association of Zambia, that wanted to work with the government on NCDs, and thus need policy direction on how to do so. The then First Lady also added to the pressure by raising the profile of cancers especially cervical cancer. Therefore, the government needed to guide the utilisation of the support it is likely to receive. An informant remarked:

“*I think we also had the figures ourselves. We saw that the cases were going up. In 2011, we had 144,000 cases of high blood pressure showing a 40% share of the NCDs. So now 144,000 cases that’s a lot. It would motivate you to do something about it… So that in itself prompted the government to act based on the statistics from hospitals and health facilities…*” (Key Informant 5)

### Policy formulation process

The key step in the policy process for the NCD strategic plan was the consultative workshop with some key stakeholders. Informants reported that the process was initiated by the MoH, which conducted the needs assessment, conceptualisation and review of critical literature and especially of health facility data. This initial step provided information for the agenda of the consultative workshop. The aim of the consultative workshop was to develop a draft proposal with input from a wide range of stakeholders. Participants in this workshop included cooperating partners like the Swedish International Development Agency, the WHO Zambia Office, Churches Association of Zambia, and clinical experts. After the workshop and further consultations with other government line ministries, the draft was submitted to the cabinet office for approval. An informant added on the adoption process:

“*After the approval, cabinet always writes us a specific letter to say that policy proposal so, so, so was approved and you required to take action like this and this. So when that happens, the Minister of Health had to call a meeting where he launched this policy just to signify that we have started implementing that policy*” (Key Informant 1)

### Lengthy policy process a challenge

Informants noted that, generally, the policy development process was too long, averaging about 2 years. This lengthy policy process resulted in changes in the group dynamics because of shifts in the composition of the stakeholders, potentially leading to loss of momentum, missing the window of opportunity and development of new policies when similar ones are still in draft awaiting approval. One informant felt that the political actors also had potential influence on the length of the policy development process. They cited an example where the Minister of Local Government helped push for the development of the policy banning smoking in public places. However, after the change of government, the process stalled resulting in the policy not being fully implemented. The policy process was also sometimes delayed by the lack of funds. A respondent remarked:

“*…the long process of consultation whereby you hold this meeting, the next time you find there are different stakeholders and they have different expectations also…*” (Key Informant 4)

“*At the same time, there must be time lines given. You can have a policy in draft form for 20 years and sometimes you start formulating a policy not knowing that another policy was already formulated a few years ago, it happens!*” (Key Informant 7)

### Actor involvement in the policy development

The major actors in the development of the NCD strategic plan was the government through the MoH. The MoH set the agenda and adopted the final document. Actors drawn from among the stakeholders in the health sector were mainly involved during the consultative workshop where the draft policy was developed. The majority of the respondents described the stakeholder participation in the consultative workshops as being active:

“*They broke into groups, thematic group each one of them looking at a certain theme and then and they came up with proposals. They will come, identify the problem and then propose what strategies/objectives. From the group work we came back to plenary, people present and then its discussed, until the draft was done, which again through further workshop and also through circulation of the draft to identified key players institutional as well as individuals who are known to be key then they came back and did a feedback*” (Key Informant 8)

The major actors who were identified in the NCD policy process included government ministries like Ministry of Community Development, Mother and Child Health, the civil society organisations like WHO, non-governmental organisations like the Diabetic Association of Zambia, representatives from the general population, Academia, subject experts, and consultants. However, some informants reported that there were still some key actors who did not participate in the policy development, a situation they feared may have implications on the policy implementation process.

“*…in this country we have so many partners who are also actors. We have our own institutions that are actors, e.g. University Teaching Hospital, is a major actor, University of Zambia, so the actors are many, I see the list to be endless. The only problem we have, I think we have underutilization of these actors*” (Key Informant 4)

### Roles of actors in the policy development

The main roles of the actors that were identified by the informants included advocacy, evidence generation, consultancy, expertise and funding. WHO, for instance, was reported to have assisted with the consultants on how this strategic plan can be designed, developed and be made useful to the country. The Diabetic Association was identified to be working with the MoH beyond policy development to implementation of activities such as training of health workers and awareness campaigns

“*Yeah, for the government, government was the leading agency. Then we had advisors like the WHO were playing an advisory role. Then we had other people like these I have mentioned the subject specialists, they brought a lot of knowledge to the table because they had first-hand experience of these things, yeah. Of course we had people from NGOs who were also very influential, especially….Zambia Heart foundation and these people dealing with diabetes….because they were always present and they used to come with ideas, how we can move about, go around this issue. So each one played their role*” (Key Informant 1)

Most of the informants noted that the Ministry itself exerted the most influence in the process. An informant from the government said this was because the government was eager to have a document in place to provide vision and to be used for comparison with other countries in the region and the world at large. Others whom some informants felt had strong influence on the process were the clinicians who had hands on experience of the impact of NCDs from the clinical setting and WHO. Commenting on policy actors, one informant explained:

“*…Everybody is an actor… So we are talking about MoH…the MCDMCH… We are talking about private sector: the private sector from the pharmaceutical side, from the clinical medical side. You are talking about civil society and when you say civil society it does not mean they sit on the round table maybe it may be pressure group. For instance, you have people living with HIV/AIDS they may advocate for a certain product and they have evidence and everything, they are also actors. You have the politicians, they are also actors. So in a nut shell also the academia they are also actors. So in terms of actors it’s a multi-disciplinary approach*” (Key Informant 6)

### Contents and focus of the NCD policies

From the document review, it was observed that the interventions in the NCD strategic plan aimed at reducing NCDs occasioned mortality in Zambia by 25% by 2025. In addition, the interventions also aimed at attaining the other eight targets listed in the Global Action Plan 2013–2020 for the prevention and control of NCDs. The common NCDs in Zambia listed in the NCD strategic plan included chronic respiratory diseases, CVD, type 2 diabetes mellitus, cancers, epilepsy, mental illnesses, oral health, eye diseases, injuries (mostly due to road traffic accidents and burns), and sickle cell anaemia. These contributed significantly to the morbidity and mortality arising from NCDs.

The content in the policy documents reviewed were oriented towards the prevention and control of NCDs. The key policy measures included strengthening the evidence base to inform the appropriate design of programs addressing NCDs; strengthening prevention, treatment, care and support services for NCDs; strengthening and scale-up of public awareness on NCDs at all levels; and strengthening ambulatory and referral systems. Building on the key policy measures, the strategic direction was system oriented with focus on prevention of NCDs and capacity building of the six building blocks of the health system to increase the access and quality of services of emerging and existing NCDs. The NCD strategic plan also recommended interventions for the reduction of the four common behavioural risk factors with focus on healthy lifestyles, primary prevention, screening and early diagnosis.

### Gaps in the policy content

#### Lack of representative and baseline data

The mid-term review of the health sector performance revealed that there was a lack of reliable data on NCDs and the prevalence of the NCDs that have been prioritised was uncertain. Most of the informants also felt that there was inadequate local data to better guide the policy content. This lack of data was reflected in the results framework of the NCD strategic plan, where some activities like reducing salt intake and increasing physical activity, had no baseline data. The lack of data further meant that the situation analysis provided in the policy documents was poor, making it difficult to set the measurable indicators and targets as pointed out by some informants. Informants further added that studies which have been conducted to generate data on NCDs and associated risk factors like the WHO STEPwise approach to surveillance (STEPs) were not representative for the country. Review of the NCD strategic plan also showed that NCDs like epilepsy, sickle cell disease, asthma and mental conditions, which according to health facility data are common, had no population or health facility prevalence data available.

Informants proposed that one way of addressing the problem of lack of baseline data on NCDs was proper utilisation of the available data collected routinely. This data could then be analysed for evidence to guide decision making.

“*But also in general, I think you look at for example if you look at Health Management Information System (HMIS) data which is what we did some time back, evidence is there. If one wants to look at HMIS data like what we did, we looked at HMIS data for hypertension and diabetes and the finding were very interesting and they can actually guide in general where you want to put your resources*” (Key Informant 2)

#### Domesticating of guidelines

Informants stated that the 4 × 4 approach of focusing on CVDs, cancers, diabetes and chronic respiratory conditions as the four main diseases and unhealthy diets, alcohol abuse, use of tobacco products and physical inactivity as the four main preventable risk factors was not adequate in an African setting. They explained that there is still a large component of NCDs in the African context which are minor in the Western World and hence not prioritised. The informants added that conditions like mental health and sickle cell disease, which are also prevalent in Africa, ought to be prioritised in national policies through domestication of guidelines on which policies are based. An informant explained on the need for domestication of guidelines:

“*But like we are saying even in the current strategic plan, what we have tried to do with those indicators is really to see what is the reality of Zambia based on what we are doing and the prevalence and then give targets, different targets for the indicators and time frame based on what is on the ground and borrowing from the generic ones. Because the ones that we borrow from WHO are generic, we can try and adapt to look at what is the reality and what can we do*” (Key Informant 2)

It was observed from the review of the NCD strategic plan that, although Zambia had gone beyond the 4 × 4 approach to include mental illness, sickle cell disease and eye conditions in the situation analysis, the interventions in the results framework focused on combating the four ‘traditional NCDs’. The main goal of the policy of reducing NCD mortality by 25% by 2025 also remained in its generic form.

#### Health education underutilised

Some of the informants felt that the contents of NCD policies should be weighted toward health promotion, education and sensitisation. It was thought that the bulk of the resources were being spent on the curative aspect even though the benefits from prevention through risk factor reduction were well known. Some informants added that health promotion and legislation approach were good preventive strategies for NCDs, but funding for these activities still remained low. It was felt that health promotion, if utilised, would raise the awareness of NCDs, and people would be empowered to make better health choices. Health education was seen as cardinal in improving the health seeking behaviour of the people, which was still low. An informant further identified an opportunity of including health education for NCDs like sickle cell disease in premarital counselling during Voluntary Counselling and Testing. On the importance of education, one informant added:

“*…This is why education is such a vaccine to a lot of problem which predisposes society to disease. People need to be educated; this is the reason for this policy; adhering or implementing this policy is for your own good. They adhere to that policy, they see the good, then they’ll accelerate its implementation*” (Key Informant 7)

#### Conflict in risk factor reduction policies

Some informants felt that there was no agreement between the NCD policy content and other government pronouncements. They felt that recent government pronouncements were undoing that which the same government had put in place to address NCDs. Despite risk factor reduction being one of the goals of the NCD policies, the government in the 2016 budget announced that it had reduced the tax on the importation of clear alcohol, which is a known risk factor for NCDs. This reduction in tax was also against the evidence that increasing taxes on alcohol and tobacco have been effective in the prevention and control of NCDs in other countries. An informant explained:

“*So it’s very tricky and complex and we really have to involve all the stakeholders so that we are really agreeing on what we want as a country, we have to speak in the same language, we can’t be talking about NCD control and then on the other hand we are supporting the same risk factors that we are supposed to be trying to mitigate*” (Key Informant 6)

## Discussion

The policy development process of the NCD strategic plan that provides the road map for addressing NCDs in Zambia was initiated by the Government itself through the MoH. This plan was developed in response to local evidence showing the rising burden of NCDs and the push from the global commitments such as the UN political declaration on NCDs. Stakeholder participation in the process was reported to be good, although some informants felt that the actors were underutilised. Although the strategic plan identified a number of NCDs as being prevalent in Zambia, including epilepsy and sickle cell disease, the plan only details interventions for the traditional NCDs, namely cancers, CVD, diabetes and chronic respiratory conditions in line with the generic 4 × 4 approach. These findings therefore highlight the strengths and challenges in the policy process of developing the NCD strategic plan.

The key elements that contributed to the successful development of the NCD strategic plan were stakeholder participation and consultation, strong political will from the government and use of international guidelines. These factors, which have been reported in other studies [25], are essential in the development of policies for NCDs because they may improve adoption and implementation of policies [26, 27]. The strong political will exhibited for the development of the NCD strategic plan could have been as a result of the international commitments that Zambia signed to such as the Political Declaration of 2011 and local evidence of an emerging problem similar to what has been observed in other countries [19, 28, 29].

While some countries have had a lack of stakeholder participation in NCD policy development process [30], this was not a problem in Zambia. The stakeholder engagement during the process took the form of a consultative workshop where key players in the health sector developed the draft of the NCD strategic plan to ensure that the resulting document was comprehensive. Studies in other countries have stressed the importance of such key implementing partners’ support in policy development [31–33].

Findings from this study show that the main challenges in the development of the NCD strategic plan was the lack of population-based data for some of the prioritised NCDs. The policy process largely depended on data from health facilities collected through the HMIS, which has limited indicators for NCDs [21]. The policy process should ideally have been preceded by a population-based survey to understand the intricate drivers of NCDs in the various cultural and geographic diversities of Zambia as did other countries [18, 20, 34]. Having baseline data on the common NCDs in Zambia would also help the government lobby for support and collaborations from stakeholders to successfully address aspects of NCDs and their risk factors, which might result in increased funding for NCD activities.

Another weakness from the policy process was the inadequate domestication of international guidelines. This study showed that the development of the NCD strategic plan was largely influenced by international agencies like WHO and, as a result, the interventions and strategic directions were based on the WHO Global Action Plan for NCDs 2013–2030 and the 4 × 4 approach. Several studies in developing countries have shown how international donors and multilateral agencies like WHO can influence health policy agendas on issues like malaria [35], maternal health [36, 37] and childhood vaccination [38]. The inadequate domestication resulted in the NCD strategic plan focusing on the four ‘traditional’ NCDs as identified by WHO [39] and without interventions for conditions like mental illness, epilepsy, eye conditions, injuries and sickle cell disease, which are common in Zambia according to health facility data. Rani et al. [29] warn that, without effectively adapting generic guidelines to local disease burden and resources, the targets and interventions for NCD risk are unclear, difficult to measure and inappropriate. There is therefore a need to utilise the complementary and more comprehensive frameworks that have been proposed for addressing NCDs in LMICs like Zambia [40].

Informants also identified that there was underutilisation of potential actors in the policy process. Although the policy process had stakeholder participation, these stakeholders were mainly health sector based, with the government driving the policy agenda. Studies have highlighted that non-health actors also have played critical roles in addressing NCDs, including enforcement of regulations and raising awareness [18, 20, 33, 41]. The 2011 Political Declaration on NCDs strongly advocated for a multi-sectoral approach to NCD policymaking because of the multifaceted origins of some NCDs [13]. The government therefore needs to begin engaging non-health actors like the media and local government authorities in the implementation of the NCD strategic plan.

The findings in this study thus imply that the NCD strategic plan in its current state is inadequate for the Zambia setting. The lack of representative data and inadequate domestication of guidelines contributed to the NCD strategic plan having gaps in its contents. The stakeholder consultation and use of evidence from the HMIS to support policy development, which was identified in this study, is a good start. However, there is a need for the government and stakeholders to invest in tools and systems that will generate representative data to guide the development of comprehensive and relevant policies. The government also needs to strengthen linkages with partners and stakeholders for the implementation of the NCD strategic plan.

### Strengths and limitations of the study

The methodological approach of triangulating the data from the document review with the key informants and vice versa enhanced the trustworthiness of the findings in this study. Nevertheless, the fact that the key informants who participated in the study were the very ones that were involved in the development of the NCD policy could have introduced response bias arising from their expectations and experiences in policy process. However, since there were no major divergent views from the informant’s data and the data from the informants was comparable to that from the document review, it is unlikely that this bias occurred.

## Conclusion

Using the policy triangle framework, this study has shown that international contextual factors were important catalysts to setting the NCD agenda in Zambia. It has also revealed a policy process dominated by the government, which played the major role in agenda setting and adoption. The content of the NCD strategic plan inadequately addressed all the major NCDs that are prevalent in Zambia as the plan was strongly skewed towards the 4 × 4 approach. Going forward, there is a need to adequately domesticate international frameworks adopted to guide policy development to match the disease burden, resources and capacities in the local context if policy measures are to be comprehensive, relevant and measurable.

This study provided information useful for advocating and improving the policy development process. This study also laid ground for areas of further exploration or evaluation such as the extent of implementation and the effectiveness of the NCD policy in achieving the goal of reducing the incidence and prevalence of NCDs in Zambia.

## Abbreviations

CVD: cardiovascular diseases
HMIS: health management and information system
LMIC: low- and middle-income countries
MCDMCH: Ministry of Community Development Mother and Child Health
MoH: Ministry of Health
NCD: non-communicable disease
NHSP: National Health Strategic Plan
WHO FCTC: World Health Organization Framework Convention for Tobacco Control

## References

[1] World Health Organization (2005). Preventing Chronic Disease: A Vital Investment.

[2] World Health Organization (2011). Global Status Report on Non-communicable Diseases 2010.

[3] Wagner K, Brath H (2012). A global view on the development of non communicable diseases. Prev Med.

[4] World Health Organization (2014). Noncommunicable diseases country profiles 2014.

[5] Mulenga D, Siziya S, Rudatsikira E, Mukonka VM, Babaniyi O, Songolo P, Muula AS (2013). District specific correlates for hypertension in Kaoma and Kasama rural districts of Zambia. Rural Remote Health.

[6] Siziya S, Rudatsikira E, Babaniyi O, Songolo P, Mulenga D, Muula A. Prevalence and correlates of hypertension among adults aged 25 years or older in a mining town of Kitwe, Zambia. J Hypertens. 2012;1(105):2167–1095.1000105.

[7] Goma FM, Nzala SH, Babaniyi O, Songolo P, Zyaambo C, Rudatsikira E, Siziya S, Muula AS (2011). Prevalence of hypertension and its correlates in Lusaka urban district of Zambia: a population based survey. Int Arch Med..

[8] Nsakashalo-Senkwe M, Siziya S, Goma FM, Songolo P, Mukonka V, Babaniyi O (2011). Combined prevalence of impaired glucose level or diabetes and its correlates in Lusaka urban district, Zambia: a population based survey. Int Arch Med.

[9] Bailey SL, Ayles H, Beyers N, Godfrey-Faussett P, Muyoyeta M, du Toit E, Yudkin JS, Floyd S (2016). Diabetes mellitus in Zambia and the Western Cape province of South Africa: Prevalence, risk factors, diagnosis and management. Diabetes Res Clin Pract..

[10] World Health Organization. Global Diabetes Report 2016: Country Profiles. http://www.who.int/diabetes/country-profiles/zmb_en.pdf. Accessed 18 Mar 2017.

[11] World Health Organization (2011). Global Status Report on Non-communicable Diseases.

[12] World Health Organization. Non-communicable Diseases and Mental Health Tools. http://www.who.int/nmh/ncd-tools/en/. Accessed 23 Mar 2016.

[13] United Nations (2011). Political Declaration of the High-level Meeting of the General Assembly on the Prevention and Control of Non-communicable Diseases.

[14] Mendis S (2010). The policy agenda for prevention and control of non-communicable diseases. 08/11/2010th ed.

[15] Mendis S, Chestnov O (2013). Policy reform to realize the commitments of the Political Declaration on noncommunicable diseases. Br Med Bull..

[16] World Health Organization (2012). Assessing national capacity for the prevention and control of noncommunicable diseases: report of the 2010 global survey.

[17] Metta E, Msambichaka B, Mwangome M, Nyato DJ, Dieleman M, Haisma H, Klatser P, Geubbels E (2014). Public policy, health system, and community actions against illness as platforms for response to NCDs in Tanzania: a narrative review. Global Health Action..

[18] Silva-Matos C, Beran D (2012). Non-communicable diseases in Mozambique: risk factors, burden, response and outcomes to date. Glob Health.

[19] Bosu WK (2012). A comprehensive review of the policy and programmatic response to chronic non-communicable disease in Ghana. Ghana Med J.

[20] Echouffo-Tcheugui J, Kengne A (2011). Chronic non-communicable diseases in Cameroon - burden, determinants and current policies. Glob Health.

[21] Aantjes CJ, Quinlan TK, Bunders JF (2014). Practicalities and challenges in re-orienting the health system in Zambia for treating chronic conditions. BMC Health Serv Res..

[22] Walt G, Gilson L (1994). Reforming the health sector in developing countries: the central role of policy analysis. Health Policy Plan.

[23] Walt G, Shiffman J, Schneider H, Murray SF, Brugha R, Gilson L (2008). ‘Doing’ health policy analysis: methodological and conceptual reflections and challenges. Health Policy Plan..

[24] Peirson L, Ciliska D, Dobbins M, Mowat D (2012). Building capacity for evidence informed decision making in public health: a case study of organizational change. BMC Public Health.

[25] Etiaba E, Uguru N, Ebenso B, Russo G, Ezumah N, Uzochukwu B, Onwujekwe O (2015). Development of oral health policy in Nigeria: an analysis of the role of context, actors and policy process. BMC Oral Health..

[26] Beaglehole R, Bonita R, Horton R, Adams C, Alleyne G, Asaria P, Baugh V, Bekedam H, Billo N, Casswell S (2011). Priority actions for the non-communicable disease crisis. Lancet.

[27] Alwan A (2010). Monitoring and surveillance of chronic non-communicable diseases: progress and capacity in high-burden countries. Lancet (London, England).

[28] Bhandari G, Angdembe M, Dhimal M, Neupane S, Bhusal C (2014). State of non-communicable diseases in Nepal. BMC Public Health..

[29] Rani M, Nusrat S, Hawken L (2012). A qualitative study of governance of evolving response to non-communicable diseases in low-and middle- income countries: current status, risks and options. BMC Public Health..

[30] Faraji O, Etemad K, Akbari Sari A, Ravaghi H (2015). Policies and programs for prevention and control of diabetes in Iran: a document analysis. Global J Health Sci.

[31] Chimeddamba O, Peeters A, Walls HL, Joyce C (2015). Noncommunicable disease prevention and control in Mongolia: a policy analysis. BMC Public Health..

[32] Odoch WD, Kabali K, Ankunda R, Zulu JM, Tetui M (2015). Introduction of male circumcision for HIV prevention in Uganda: analysis of the policy process. Health Res Policy Syst..

[33] Al-Bahlani S, Mabry R (2014). Preventing non-communicable disease in Oman, a legislative review. Health Promot Int..

[34] Islam A, Biswas T (2014). Chronic non-communicable diseases and the healthcare system in Bangladesh: current status and way forward. Chronic Dis Int.

[35] Woelk G, Daniels K, Cliff J, Lewin S, Sevene E, Fernandes B, Mariano A, Matinhure S, Oxman AD, Lavis JN (2009). Translating research into policy: lessons learned from eclampsia treatment and malaria control in three southern African countries. Health Res Policy Syst..

[36] Koduah A, van Dijk H, Agyepong IA (2015). The role of policy actors and contextual factors in policy agenda setting and formulation: maternal fee exemption policies in Ghana over four and a half decades. Health Res Policy Syst..

[37] Deleye C, Lang A (2014). Maternal health development programs: comparing priorities of bilateral and private donors. BMC Int Health Human Rights..

[38] Chilengi R, Rudd C, Bolton C, Guffey B, Masumbu PK, Stringer J (2015). Successes, challenges and lessons learned in accelerating introduction of rotavirus immunisation in Zambia. World J Vaccin.

[39] World Health Organization (2013). Global action plan for the prevention and control of noncommunicable diseases 2013-2020.

[40] Binagwaho A, Muhimpundu MA, Bukhman G (2014). 80 under 40 by 2020: an equity agenda for NCDs and injuries. Lancet.

[41] Tantivess S, Walt G (2008). The role of state and non-state actors in the policy process: the contribution of policy networks to the scale-up of antiretroviral therapy in Thailand. Health Policy Plan.

